# Exploring a crossroads in the aging process

**DOI:** 10.7554/eLife.109320

**Published:** 2025-10-30

**Authors:** Kiyan Shabestary, Daniel E Gottschling

**Affiliations:** 1 Calico Life Sciences LLC San Francisco United States

**Keywords:** aneuploidy, mitosis, aging, intron, RNA, NPC, *S. cerevisiae*

## Abstract

Experiments on yeast show that the nuclear pore complex has a central role in the loss of chromosomes associated with aging.

**Related research article** Mirkovic M, McCarthy J, Meinema AC, Parenteau J, Lee SS, Elela SA, Barral Y. 2025. Dissociation of the nuclear basket triggers chromosome loss in aging yeast. *eLife* **14**:RP104530. doi: 10.7554/eLife.104530.

Living systems are a bit like the traffic in a city: when the roads are repaired on a regular basis, the traffic flows. But if the roads are not maintained, wear and tear accumulates, disruptions spread, and traffic begins to grind to a halt. Aging is much the same. It progresses through the accumulation of small molecular defects that gradually build up to a point where an organism can no longer function, ultimately leading to its death. A central goal of aging research is to trace this cascade and understand how specific molecular failures escalate into systemic dysfunction.

In the model organism *Saccharomyces cerevisiae* (budding yeast), several distinct aging phenotypes have been described, including vacuolar alkalinization, mitochondrial dysfunction, and genomic instability (Shabestary et al., 2025). Although researchers do not fully understand how these phenomena combine to create a cascade of cellular decline, there has been progress in connecting another phenomenon – the accumulation of structures called extrachromosomal rDNA circles (ERCs; Sinclair and Guarente, 1997) – to such a cascade.

ERCs exist in a dynamic balance with the highly repetitive ribosomal DNA (rDNA) chromosome locus from which they originate (Hori et al., 2023). Beyond a certain threshold, the accumulation of ERCs appears to be amplified exponentially, leading to a state of broader functional decline marked by an abrupt late-life slowdown in the rate of cell division (Morlot et al., 2019). Several hypotheses have been proposed to explain how this happens. One idea is that excess ERCs can deplete a limited pool of chromosome-maintenance factors (Feser et al., 2010; Ganley and Kobayashi, 2014; Kobayashi et al., 1998). Another idea – proposed by Yves Barral (ETH Zürich) and colleagues – is that ERCs shorten lifespan by tethering to the nuclear pore complex, which is the main gateway in and out of the nucleus in cells (Meinema et al., 2022). However, it is not clear how these processes contribute to functional decline.

Now, in eLife, Barral and co-workers at ETH Zürich and the Université de Sherbrooke – including Mihailo Mirkovic as first author – report how they have built on their earlier work to provide evidence for a possible link between ERC-mediated disruption of the nuclear pore complex and genomic instability (Mirkovic et al., 2025). In addition to possibly explaining how the instability of ribosomal DNA – an instability that allows ERCs to form – drives system-wide decline in old yeast cells, the work also highlights the nuclear pore complex as a potential crossroads or nexus linking ERCs to other aging phenomena (Figure 1).

**Figure 1.**
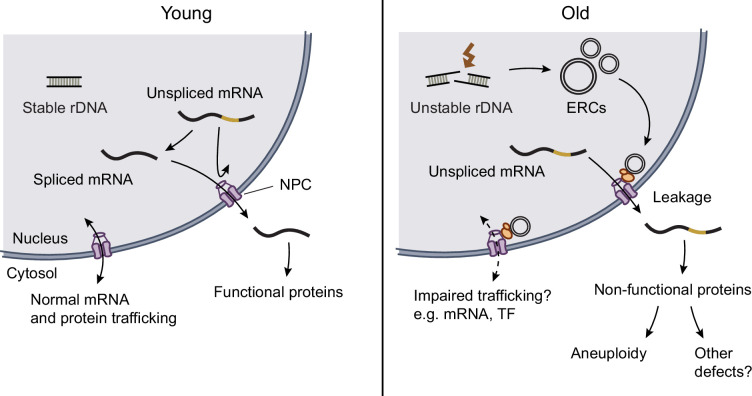
How the accumulation of ERCs might lead to aneuploidy in aged cells. In young yeast cells (left), ribosomal DNA (rDNA) is stable, unspliced messenger RNA (mRNA) is confined to the nucleus (grey region), and nuclear pore complexes allow spliced mRNAs to leave the nucleus (so that they can be translated to produce functional proteins). These complexes also allow various proteins, such as transcription factors, to enter and leave the nucleus. In old yeast cells (right), ribosomal DNA is unstable, leading to the formation of extrachromosomal rDNA circles (ERCs), which are tethered to nuclear pore complexes by SAGA complexes (orange). This disrupts the function of the nuclear pore complexes. One consequence of this is that unspliced mRNAs can inappropriately leave the nucleus, leading to the formation of non-functional proteins, including non-functional forms of proteins that are involved in chromosome segregation, which can then lead to cells having the wrong number of chromosomes after cell division (aneuploidy) and, possibly, other defects. SAGA: Spt-Ada-Gcn5 acetyltransferase; TF: transcription factor.

Many aged yeast cells arrest and die in S-phase, indicating a problem initiating or completing DNA replication (Neurohr et al., 2018). Using a fluorescent reporter for chromosome localization, Mirkovic et al. first revisited this observation and found that old yeast cells indeed experience genomic instability and lose chromosomes (aneuploidy). They further showed that chromosome mis-segregation could be modulated using mutants with increased or decreased levels of ERCs, thus implicating the accumulation of ERCs in this aging process.

The missing link at this stage was how ERC accumulation drives chromosomal mis-segregation. A clue came from the observation that three genes that encode for proteins that are needed to ensure proper chromosome segregation all contain introns that implicate ERC accumulation in the processing and/or export of messenger RNA. This was validated by showing that unspliced messenger RNAs increasingly leak from the nucleus into the cytoplasm during aging, but not in mutants with reduced ERC formation. Removing the introns from those three genes rescued mis-segregation in old cells. While the mechanistic connection between disruption of the nuclear pore complex and the leakage of unspliced messenger RNA leakage remains to be mechanistically demonstrated, this work nevertheless suggests the nuclear pore complex as a likely point of convergence between ERC accumulation and genomic instability.

Together, these findings highlight important weaknesses that could lead to aging. First, the presence of introns in a gene could increase the chance of a functional imbalance in the relevant protein in old cells. It is worth noting that the genes for most ribosomal proteins contain introns, so these proteins could be compromised later in life (Ares et al., 1999). Second, nuclear pore complexes contain long-lived proteins with very low turnover (Rempel et al., 2019), making them particularly vulnerable to damage and dysfunction, as they cannot be readily replaced. Third, nuclear pore complexes are also involved in protein trafficking, which suggests that transcription- or splicing-factor-based gene regulation could also be impacted with age (Rempel et al., 2019).

Although ERCs are specific to budding yeast, the results of Mirkovic et al. could have implications that extend well beyond this organism. Nuclear pore complexes are long-lived structures with a conserved role in mRNA and protein trafficking, and show little turnover in post-mitotic cells, so similar vulnerabilities may underlie aging in animals, where introns are much more common than they are in yeast (D’Angelo et al., 2009; Vasu et al., 2001; Wente, 2000). Indeed, it is known that the nuclear pore complex is a key driver of aging in mammals, which suggests that dysfunctions not mediated by ERCs might also converge on this complex (Cho and Hetzer, 2020). Together, these findings suggest that NPC-mediated trafficking is a conserved weak link in aging. Much like the traffic lights in a city, nuclear pore complexes quietly orchestrate the flow of molecular traffic, and when they break down, the resulting gridlock can ripple through the entire system.
